# Comparison of a novel self-expanding transcatheter heart valve with two established devices for treatment of degenerated surgical aortic bioprostheses

**DOI:** 10.1007/s00392-023-02181-9

**Published:** 2023-04-05

**Authors:** Olga Nikolayevska, Lenard Conradi, Johannes Schirmer, Hermann Reichenspurner, Florian Deuschl, Stefan Blankenberg, Ulrich Schäfer

**Affiliations:** 1 Department of General and Interventional Cardiology, University Heart and Vascular Center, Klinik für Kardiologie, University Medical Center Hamburg-Eppendorf, Martinistraße 52, 20246 Hamburg, Germany; 2 Department of Cardiovascular Surgery, University Heart and Vascular Center, University Medical Center Hamburg-Eppendorf, Hamburg, Germany; 3 Department of Cardiology, Heart and Vascular Centre Bad Bevensen, Bad Bevensen, Germany

**Keywords:** TAVI, Valve-in-valve, Aortic valve disease, Bioprosthetic valves

## Abstract

**Aims:**

This study was performed to compare haemodynamic properties of a novel transcatheter heart valve (THV) with two established valve technologies for treatment of failing surgical aortic bioprosthetic valves (SAV). The ALLEGRA THV has been recently described with a proven safety and performance profile.

**Methods and results:**

The study was designed as a retrospective, single-centre study investigating 112 patients (77.7 ± 7.1 years, 53.8% female, STS score 6.8 ± 5.8% and logEuroSCORE I 27.4 ± 16.1%) with failing SAV. Patients were treated with the ALLEGRA THV (NVT, n = 24), the CoreValve/EvolutR (MTD, n = 64) or the Edwards Sapien/Sapien XT/Sapien 3 (EDW, n = 24). Adverse events, haemodynamic outcomes and patient safety were analysed according to VARC-3 definitions. Overall procedural success was high (94.6%), even though 58.9% of the treated SAV were classified as small (true inner diameter < 21 mm). After treatment, the mean pressure gradient was significantly reduced (baseline: 33.7 ± 16.5 mmHg, discharge: 18.0 ± 7.1 mmHg), with a corresponding increase in effective orifice area (EOA). The complication rates did not differ in between groups. There was a trend to lower mean transvalvular gradients after implantation of self-expanding THV with supra-annular valve function, despite a higher frequency of smaller SAVs in the NVT and MTD group. Additionally, comparison between NVT and MTD revealed statistically lower transvalvular gradients (NVT 14.9 ± 5.0 mmHg, MTD 18.7 ± 7.5 mmHg, *p* = *0.0295*) in a subgroup analysis.

**Conclusions:**

Valve-in-valve (ViV) treatment of failing SAV with supra-annular design like the ALLEGRA THV resulted in favourable haemodynamic outcomes with similar low clinical event rates and may therefore be an interesting alternative for VIV TAVI.

**Graphical abstract:**

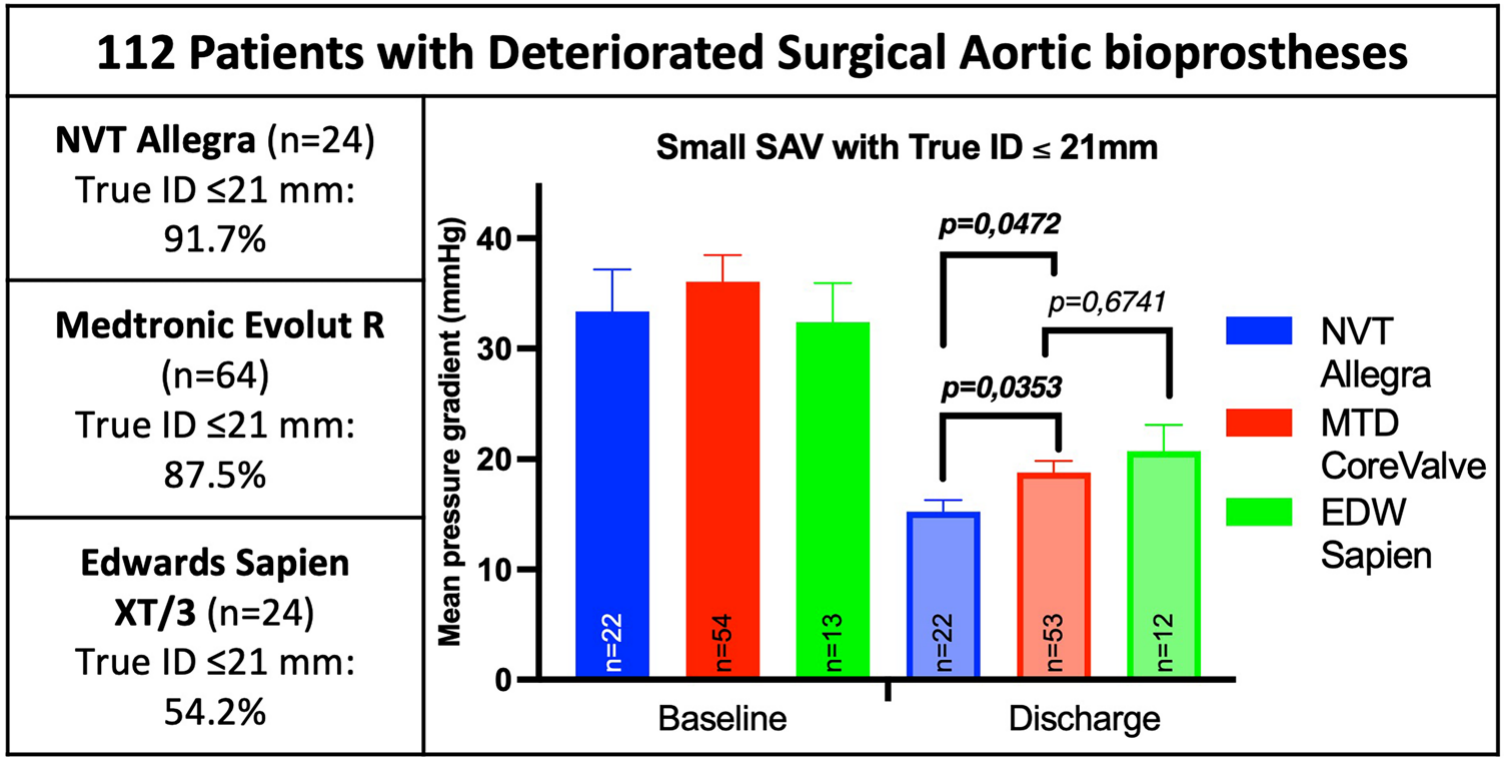

**Supplementary Information:**

The online version contains supplementary material available at 10.1007/s00392-023-02181-9.

## Introduction

Aortic valve disease represents a significant problem in elderly patients, frequently requiring valve replacement. In the past decades, surgical aortic valve replacement (SAVR) has become the preferred treatment for these patients, with mostly bioprosthetic valves being used, mainly due to the avoidance of life-long anticoagulation therapy [1, 2]. However, the durability of bioprostheses is known to be limited due to irreversible destructive processes, leading to stenosis, regurgitation, or both [3, 4].

Even though reoperation has been considered as the gold standard for treatment of degenerated surgical aortic valves (SAV) by many surgeons until recently, valve-in-valve (VIV) treatment has emerged over time and is now considered as a valid alternative to avoid redo SAVR in patients with increased surgical risk for reoperation [5, 6]. VIV utilization is less invasive and therefore associated with relatively low rates of mortality as well as major complications and with an excellent improvement in functional capacity and quality of life outcomes at 1 year [7–13]. The multi-centre VIV-trial VIVA, utilizing the transcatheter heart valves (THV) CoreValve and Evolut R (Medtronic, Minneapolis, Minnesota) confirmed a consistent safety and efficacy after VIV interventions, using a self-expanding supraannular THV for failing SAV [14]. Even SAV with small inner diameters were safely treated, albeit significantly higher gradients were evident. The later observation was previously described in substudies of registries with small SAV (label size < 21 mm), a stenotic pattern of SAV degeneration and pre-existing prosthesis-patient mismatch (PPM) of the SAV, all of them being associated with higher residual gradients, less improvements in functional capacity and increased risk of mortality following VIV treatment [12, 14–18].

Recently, a first in-human clinical study with the ALLEGRA THV (NVT/Biosensors, Hechingen, Germany), a novel self-expanding THV, as well as *in-vitro* tests showed favourable haemodynamic and hydrodynamic results, even in small SAV [19–23]. The 30-day data as well as one-year outcomes of the VIVALL study with the ALLEGRA THV were recently published and showed excellent haemodynamic outcomes, a high survival rate and an extremely low rate of paravalvular regurgitation in a patient population predominated by very small bioprostheses [24, 25].

However, little is known about the differences to commonly used THV. Therefore, the aim of this study was to assess comparative outcomes between the most frequently used THV types and their implications for clinical practice.

## Methods

This study was designed as a single-centre, retrospective analysis of patients with degenerated SAV, who received ViV from 2008 to 2018. The aim of this analysis was to compare three different THV models (CoreValve/Evolut R, Medtronic, Minneapolis, Minnesota vs. SAPIEN/SAPIEN XT/SAPIEN 3, Edwards Lifesciences, Irvine, California vs. Allegra, New Valve Technology/Biosensors GmbH, Hechingen, Germany) regarding procedural and haemodynamical outcomes. The study complies with the Declaration of Helsinki and all patients provided written informed consent prior to implantation.

### Study population

The study population comprised 112 adults with symptomatic degenerated SAV. All patients were assessed by an interdisciplinary Heart Team and found eligible for ViV implantation using the above mentioned THVs, due to increased surgical risk for reoperation.

### Study devices

The Allegra THV is a self-expanding THV with bovine pericardial leaflets attached to a nitinol stent frame in a supraannular position. A distribution of radial force enhances a safe anchoring of the prosthesis within the aortic annulus. The stent frame allows pole movement, aiming to reduce stress on the leaflets and the ventricular inner inflow section is covered by a bovine sealing skirt, to prevent paravalvular leakages.

The CoreValve is also a self-expanding THV made of bovine pericardial tissue, which is mounted and sutured in a nitinol stent frame with a supra-annular function. The stent has a special shape with a constrained middle part for preservation of the coronary flow and an expanding upper part for fixation in the ascending aorta. The Evolut R is the next generation of the CoreValve with a shorter outflow portion of the stent for better adaptation to the aortic anulus.

The SAPIEN THV is a balloon-expanding system with three-leaflet bovine pericardial tissue sewed onto a stainless-steel frame. The Edwards SAPIEN THV system was succeeded by the SAPIEN XT system, which uses a cobalt-chromium stent and a new delivery system allowing smaller sheath sizes and therefore easier transfemoral access. Afterwards, the SAPIEN 3 was introduced, which had a major change in the valve design. The new THV is taller and has an additional outer polyethylene terephthalate sealing skirt, which aims at decreasing the degree of aortic regurgitation.

### Study endpoints and assessments

Study endpoints for THV comparison were collected and analysed following the valve academic research consortium-3 (VARC-3) criteria, including procedural and haemodynamical outcomes, coronary occlusions, in-hospital mortality and permanent pacemaker requirement. All patients were clinically assessed at admission, during the procedure and prior to discharge. Haemodynamic measurements were standardized performed according to guidelines for echocardiographic evaluation of transaortic haemodynamics [5, 26].

### Statistical analysis

All recorded variables were compared with baseline values using appropriate descriptive summary statistics (continuous and ranked data: sample size, mean, standard deviation, standard error of the mean). The descriptive statistics of a variable were calculated for each defined change. Changes were calculated as differences to the pre-treatment value only. For comparisons using 2-samples either paired or unpaired Student’s t-test were performed, for 3-sample comparisons a One-Way ANOVA. Subgroup analysis of Mitroflow SAV, different failure modes and true inner diameters of the failing bioprosthetic valves were additionally performed.

## Results

### Patient characteristics

From 2008 to 2018, a total of 112 patients underwent ViV TAVI. Patient demographics are reported in Table 1. Mean age was 77.7 ± 7.1 years, 46.2% of the patients were male and most were severely symptomatic in NYHA Class III or IV (78.6%). Mean logistic EuroSCORE I and STS-Score were 27.4 ± 16.1% and 6.8 ± 5.8%, respectively. Most common comorbidities were arterial hypertension, coronary artery disease and pulmonary hypertension. The patient characteristics mostly did not differ in between groups, apart from more male patients receiving Edwards SAPIEN THV and a higher number of patients with pulmonary hypertension in the NVT Allegra group.

**Table 1:** Baseline Characteristics

	All (n = 112)	NVT Allegra (n = 24)	Medtronic CoreValve/ Evolut R (n = 64)	Edwards Sapien (n = 24)	p-value
Age (years)	77.7 ± 7.1	79.1 ± 4.4	77.9 ± 7.1	75.7 ± 8.8	*0.24*
Male (%)	52 (46.2)	9 (37.5)	22 (34.4)	21 (87.5)	** < *0.0001***
NYHA III/IV (%)	88 (78.6)	15 (62.5)	56 (87.5)	17 (70.8)	*0.09*
Coronary artery disease (%)	71 (63.4)	16 (66.7)	40 (62.5)	15 (62.5)	*0.93*
Previous PCI (%)	40 (35.7)	13 (54.2)	19 (29.7)	8 (33.3)	*0.10*
Previous Stroke (%)	21 (18.8)	6 (25.0)	12 (18.8)	3 (12.5)	*0.55*
COPD (%)	26 (23.2)	8 (33.3)	12 (18.8)	6 (25.0)	*0.35*
Peripheral artery disease (%)	31 (27.7)	8 (33.3)	19 (29.7)	4 (16.7)	*0.54*
Chronic renal insufficiency (%)	66 (58.9)	14 (58.3)	43 (67.2)	9 (37.5)	***0.04***
Diabetes (%)	18 (16.1)	5 (20.8)	10 (15.6)	3 (12.5)	*0.73*
Arterial hypertension (%)	94 (83.9)	22 (91.7)	52 (81.3)	20 (83.3)	*0.19*
Pulmonary Hypertension (%)	89 (79.5)	20 (83.3)	58 (64.7)	11 (45.8)	** < *0.0001***
Porcelain Aorta (%)	29 (25.9)	6 (25.0)	16 (25.0)	7 (29.2)	*0.78*
SAV failure mode (%)					
Stenosis	51 (45.5)	6 (25.0)	35 (54.7)	10 (41.7)	*0.06*
Insufficiency	14 (12.5)	2 (8.3)	8 (12.5)	4 (16.7)	*0.69*
Mixed	47 (42.0)	16 (66.7)	21 (32.8)	10 (41.7)	***0.016***
logEuroSCORE I (%)	27.4 ± 16.1	25.6 ± 9.8	30.1 ± 18.5	22.2 ± 13.2	*0.10*
STS Score (%)	6.8 ± 5.8	5.8 ± 3.3	7.4 ± 6.2	6.5 ± 6.8	*0.51*
SAV True ID (n)					
< 19 (mm)	28 (25.0)	6 (25.0)	19 (29.7)	3 (12.5)	*0.26*
≥ 19–20 (mm)	38 (33.9)	10 (41.7)	24 (37.5)	4 (16.7)	*0.12*
≥ 21 (mm)	46 (41.1)	8 (33.3)	21 (32.8)	17 (70.8)	***0.0032***
Time interval to index procedure (years)	9.1 ± 4.7	9.0 ± 4.5	8.6 ± 4.4	10.6 ± 5.6	*0.20*

The *p*-values are in italics and the significantly different *p*-values are highlighted as bold

### SAV characteristics

SAV dysfunction was most frequently due to isolated stenosis (45.5%) or combined stenosis and insufficiency (42.0%). The majority of the treated SAV had small true inner diameters of < 21 mm (58.9%). Mean time from index surgery to VIV was 9.1 ± 4.7 years. The most commonly treated SAV were Medtronic Hancock (28.6%), Sorin Mitroflow (27.7%) or Edwards Perimount valves (19.6%). Further average failure modes and true inner diameters of the degenerated SAVs are summarized in Table 1.

### Procedural characteristics

**Fig. 1:**
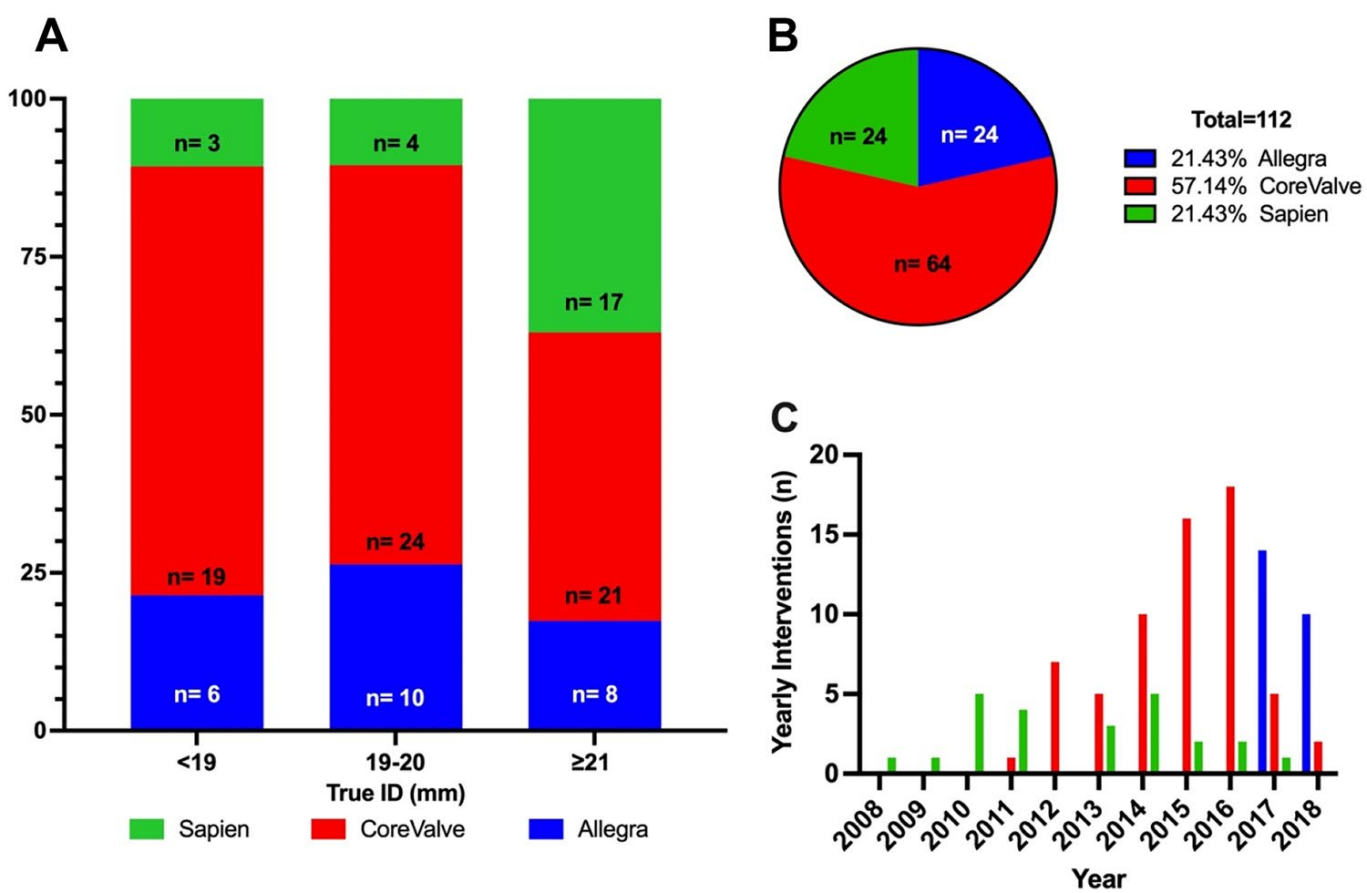
**a** Distribution of the treated SAV according to the true inner diameter. **b** Distribution of the treated SAV according to manufacturer. **c** Distribution of the yearly performed ViV-interventions in the years 2008–2018

During the first 2 years of the study period (2008–2010) only SAPIEN THV were implanted. ViV TAVI using CoreValve THV were performed since 2011 being the predominantly used THV from 2012 to 2016. In 2017, the most frequently used THV was the NVT Allegra THV (see Fig. 1).

Out of 112 patients, 106 (94.6%) were technically successfully implanted. Device success and VARC-3 data are summarized in Table 2. Most patients (77.7%) received transfemoral THV implantation. Conversely, Edwards SAPIEN THV were predominantly implanted transapically (75%). Preballooning of degenerated bioprosthetic SAV was performed in five patients, four treated during the early experience.

**Table 2:** Procedural Outcomes

	All (n = 112)	NVT Allegra (n = 26)	Medtronic CoreValve/ EvolutR (n = 63)	Edwards Sapien (n = 23)	p-value
Procedural success (%)	106 (94.6)	24 (92.3)	60 (95.2)	22 (95.7)	*0.84*
Predilatation (%)	5 (4.5)	0 (0)	4 (6.3)	1 (4.2)	*0.43*
Postdilatation (%)	51 (45.5)	19 (73.1)	30 (47.6)	2 (8.3)	** < *0.0001***
Need for second valve (%)	4 (3.6)	2 (7.7)	1 (1.6)	1 (4.2)	*0.37*
Vascular complications (VARC3) (%)					
Major	4 (3.6)	0 (0)	4 (6.3)	0 (0)	*0.20*
Minor	19 (17.0)	7 (26.9)	11 (17.5)	1 (4.2)	*0.11*
Non-vascular complications (VARC-3) (%)					
Major	3 (2.7)	0 (0)	1 (1.6)	2 (8.3)	*0.12*
Minor	0 (0)	0 (0)	0 (0)	0 (0)	*–*
Resheathing (%)	25 (22.3)	1 (4.2)	23 (36.5)	0 (0)	***0.001***
Surgical conversion (%)	0 (0)	0 (0)	0 (0.0)	0 (0)	–
Valve dislocation (%)	3 (2.7)	1 (4.2)	1 (1.6)	1 (4.3)	*0.72*
Coronary obstruction (%)	1 (0.1)	0 (0.0)	1 (1.6)	0 (0)	*0.68*
Mortality (%)	5 (4.5)	0 (0)	5 (7.9)	0 (0)	*0.13*
Permanent Pacemaker implantation (%)	13 (11.6)	2 (7.7)	9 (14.3)	2 (8.3)	*0.61*

The *p*-values are in italics and the significantly different *p*-values are highlighted as bold

Postdilation was performed most frequently in the Allegra group (73.1%), compared to CoreValve/Evolut R (47.6%) and SAPIEN groups (8.3%). Resheathing and repositioning was most frequently performed when using the CoreValve/Evolut THV (36.5%). Permanent pacemaker implantation was required in 13 patients (11.6%).

According to VARC-3 definitions, four and 19 patients experienced major (3.6%) and minor (17.0%) vascular complications, respectively. Most minor vascular complications were observed after NVT Allegra implantation (26.9%), with mainly access-related complications (mostly small vascular pseudoaneurysms). Major vascular complications occurred in four patients after CoreValve implantation. The patients experienced closure device failure, resulting in two cases of VARC type 2 and and one case of type 3 bleeding, respectively. In one case, closure device failure resulted in limb ischemia, requiring intervention and removal of the closure device.

Access-related non-vascular complications were also observed. Two transapical SAPIEN THV interventions (8.3%) resulted in major complications, due to thoracic bleeding, one with the need for surgical intervention. One (1.6%) major access-related non-vascular complication occurred after CoreValve implantation using a direct transaortic access. A venous bypass graft of the right coronary artery (RCA) was injured during suturing of the aorta, leading to haemodynamic instability and death of the patient.

In two cases, an attempted Allegra THV implantation was aborted due to a very horizontal aorta. The THV was retrieved and procedural conversion using an Evolut R was performed with good procedural outcome. Valve embolization occurred in three patients, one in every THV group. A supra-annular embolized Allegra THV was left in place and successfully treated with a subsequent Evolut R. Similarly, the cases with an embolized Evolut R and SAPIEN THV were found stable with valves sitting in the abdominal or ascending aorta, respectively. Both patients received a successful subsequent implantation of an Edwards SAPIEN valve with good haemodynamic outcomes. Among all other implantations, the intended implantation depth, with the THVs anchored a few millimetres below the sewing ring, was achieved in the vast majority of cases (data not shown).

Coronary obstruction occurred in one patient after Evolut R implantation in a deteriorated 23 mm Mitroflow valve and resulted in stenting of the left coronary artery with CPR and emergency ECMO implantation with fatal outcome. Overall in-hospital mortality was 4.5% (all cases occurring after CoreValve/Evolut R implantation). Besides coronary obstruction, other causes of death were CPR and right heart failure after accidental injury of the RCA venous-bypass graft, sepsis and multiple organ failure due to endocarditis and aortic root abscess formation in one case, as well as nosocomial pneumonia in another case. Last but not least, one patient developed a sepsis with unknown origin of infection.

### Haemodynamic outcomes

**Fig. 2:**
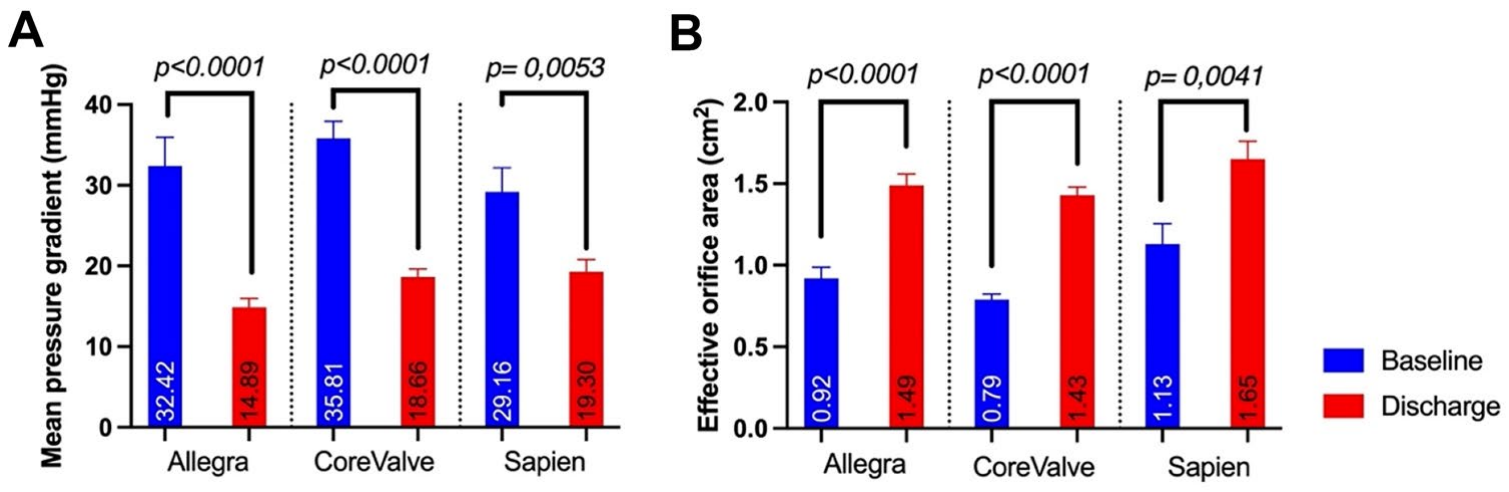
**a** Echocardiographic mean aortic pressure gradient before and after ViV-TAVI with the NVT Allegra, Medtronic CoreValve/Evolut and Edwards SAPIEN THV. b Echocardiographic effective orifice area of the aortic valve before and after ViV-TAVI with the NVT Allegra, Medtronic CoreValve/Evolut and Edwards SAPIEN THV

Echocardiographic measurements demonstrated a consistent decrease in mean pressure gradients with a corresponding increase in effective orifice area after treatment with all three THV (see Fig. 2a, b). The lowest mean pressure gradients (14.9 ± 5.0 mmHg) as well as the largest decrease of mean pressure gradients from baseline to discharge were found after VIV with the NVT Allegra THV. Additionally, the largest increase in effective orifice area (Δ = 0.57 cm^2^) was found after NVT Allegra valve implantation. However, the largest effective orifice area was observed after implantation of Edwards SAPIEN THV (1.65 cm^2^ ± 0.4), due to mostly larger treated SAV. A detailed summary of the haemodynamic outcomes can be found in Table 3.

**Table 3:** Haemodynamical Outcomes

	All (n = 112)	NVT Allegra (n = 24)	Medtronic CoreValve/ Evolut R (n = 64)	Edwards Sapien (n = 24)	p-value
Pmean Discharge (mmHg)	18.0 ± 7.1	14.9 ± 5.0	18.7 ± 7.5	19.30 ± 7.0	*0.06*
Pmax Discharge (mmHg)	33.9 ± 13.0	28.9 ± 9.7	35.2 ± 14.3	35.5 ± 11.0	*0.11*
Invasive measurement (mmHg)					
Pmean	12.6 ± 5.3	11.4 ± 3.0	12.3 ± 4.7	18.7 ± 10.2	***0.004***
Pmax	7.3 ± 6.4	4.8 ± 4.2	8.6 ± 6.2	7.4 ± 11.0	*0.07*
EOA Discharge (cm^2^)	1.52 ± 0.4	1.49 ± 0.3	1.47 ± 0.4	1.65 ± 0.4	*0.12*
Indexed EOA Discharge (m^2^/cm^2^)	0.83 ± 0.3	0.84 ± 0.2	0.81 ± 0.3	0.88 ± 0.3	*0.62*
Pmean discharge (mmHg)					
Stenosis	18.9 ± 6.1	15.3 ± 3.7	19.0 ± 6.4	20.6 ± 6.0	*0.27*
Regurgitation	14.9 ± 7.5	11.5 ± 10.6	12.1 ± 5.6	22.1 ± 7.6	*0.08*
Combined	18.0 ± 7.6	15.2 ± 5.2	20.7 ± 8.7	16.8 ± 7.6	*0.10*
EOA discharge (cm^2^)					
Stenosis	1.43 ± 0.3	1.48 ± 0.2	1.40 ± 0.4	1.48 ± 0.3	*0.82*
Regurgitation	1.48 ± 0.4	1.25 ± 0.1	1.47 ± 0.3	1.60 ± 0.5	*0.58*
Combined	1.54 ± 0.4	1.52 ± 0.4	1.41 ± 0.3	1.85 ± 0.4	*0.66*
Pmean discharge True ID (mmHg)					
< 19 mm	19.5 ± 7.2	15.8 ± 3.9	19.4 ± 7.3	27.7 ± 7.0	*0.06*
19–20 mm	18.3 ± 7.8	15.3 ± 6.3	19.2 ± 8.4	22.3 ± 5.7	*0.28*
≥ 21 mm	16.7 ± 6.3	13.6 ± 4.6	17.4 ± 6.9	17.2 ± 6.1	*0.37*
EOA discharge True ID (cm^2^)					
< 19 mm	1.26 ± 0.3	1.32 ± 0.3	1.25 ± 0.3	1.10 ± 0.0	*0.76*
19–20 mm	1.44 ± 0.3	1.51 ± 0.3	1.40 ± 0.3	1.37 ± 0.4	*0.59*
≥ 21 mm	1.64 ± 0.4	1.59 ± 0.4	1.57 ± 0.4	1.77 ± 0.4	*0.42*

The *p*-values are in italics and the significantly different *p*-values are highlighted as bold

**Fig. 3:**
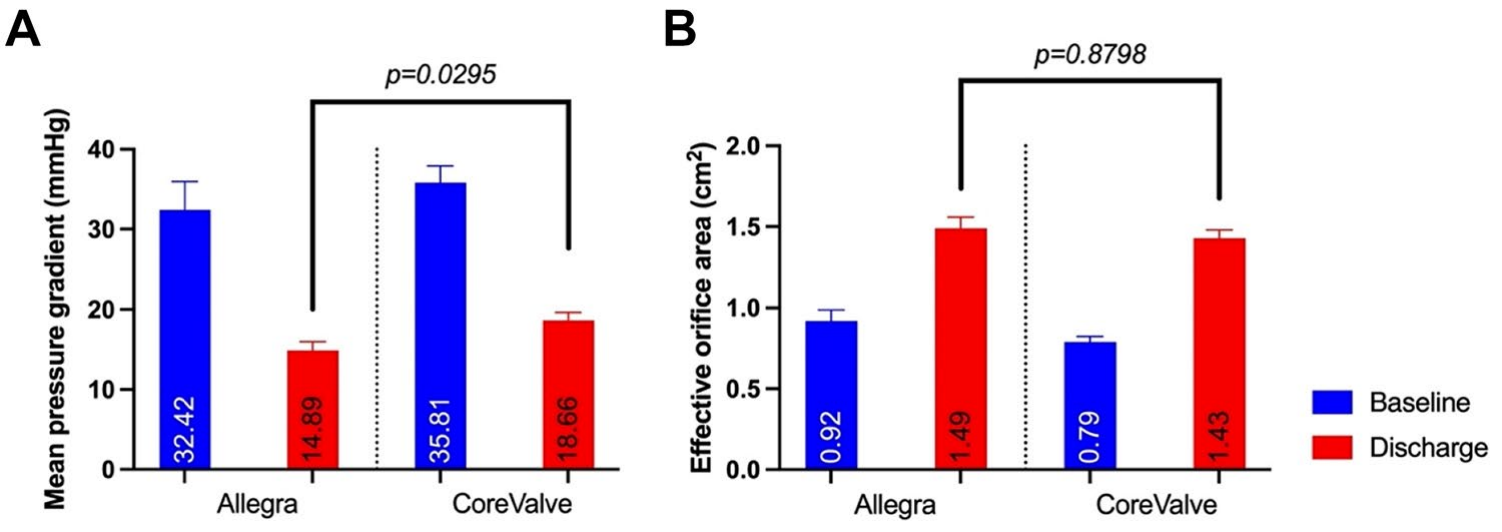
**a** Echocardiographic mean aortic pressure gradient before and after ViV-TAVI with the NVT Allegra and MTD CoreValve/Evolut. **b** Echocardiographic effective orifice area before and after ViV-TAVI with the NVT Allegra and MTD CoreValve/Evolut

**Fig. 4:**
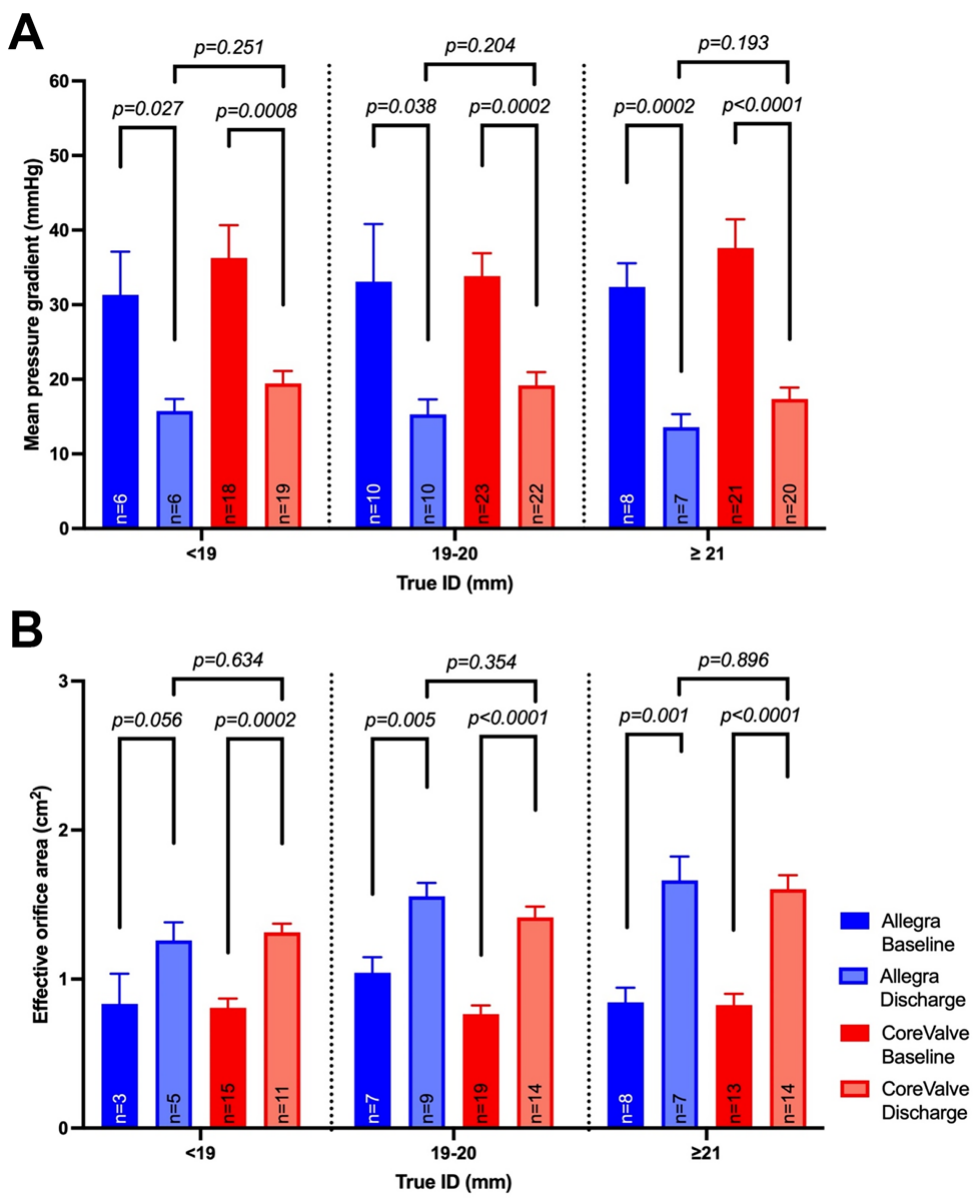
**a** Comparison of mean pressure gradients before and after ViV TAVI with NVT Allegra and MTD CoreValve according to the true inner diameter of the SAV. **b** Comparison of the effective orifice area before and after ViV TAVI with the NVT Allegra and MTD CoreValve according to the true inner diameter of the SAV

The direct comparison of the two supraannular THVs, NVT Allegra and MTR CoreValve/Evolut R showed favourable haemodynamic results for the NVT Allegra THV (NVT: 14.9 ± 5.0 mmHg, MTR: 18.7 ± 7.5 mmHg, *p* = *0.0295*) independently of the true inner diameter (True ID) of the SAV (see Fig. 3 and Fig. 4). Additional analysis of the underlying SAV failure modes, also revealed significantly lower mean pressure gradients in mixed aortic valve disease and mostly larger EOAs (data not shown) after ViV TAVI with the NVT Allegra THV, aside from regurgitant SAVs. (see supplement Fig. 1). In addition, ViV implantation in deteriorated Sorin Mitroflow SAV showed a significant decrease in the mean pressure gradient and an increase in the EOA for both THV with supraannular valve function. The results are summarized in supplement Fig. 2, demonstrating numerically favourable haemodynamic outcomes after NVT Allegra THV implantation, albeit not reaching statistical significance.

## Discussion

This study compares three types of THV for VIV TAVI, including two supraannular and one intraannular THV. Each THV showed satisfactory early haemodynamic outcomes with low early mortality and complication rates, suggesting that all three valve types are safe and feasible for use in degenerated SAV.

The main finding of our study was, that at discharge, lowest mean pressure gradients were observed after ViV TAVI with the NVT Allegra THV and largest effective orifice areas after Edwards SAPIEN implantation. It is worth noting, that most of the patients in the SAPIEN group received treatment of large SAVs with significantly larger inner diameters. Conversely, patients receiving supraannular valves represented rather challenging study populations, consisting mostly of patients with small SAVs, in addition to a large proportion of patients with Sorin Mitroflow bioprostheses. Earlier studies have shown a higher risk for coronary obstruction in patients with Mitroflow SAV, due to externally mounted leaflets, resulting in higher mortalities after ViV TAVI [27, 28]. In the NVT Allegra subgroup, the proportion of Mitroflow valves was the highest with no safety related outcomes compared to the Medtronic CoreValve/Evolut group demonstrating two serious adverse events with CPR (n = 2) and coronary obstruction in one patient.

Results from the VIVID registry have also identified a stenotic pattern of bioprostheses as a risk factor for an elevated mean pressure gradient and higher mortality after ViV treatment [15]. A similar observation was found in our data with lower gradients in pure regurgitant SAV. The difference might be best explained by more aggressive treatment of pannus with a balloon expandable THV. Interestingly, NVT valves demonstrated numerically the lowest gradients in this sub-analysis. The highest rate on postdilatations were performed in the NVT group (73.1%), potentially leading to lower transvalvular gradients [29]. Despite better haemodynamic results after ViV TAVI with the Allegra THV, there was no statistical difference to the EOAs and indexed EOAs. This observation emphasizes the fact, that an isolated interpretation of valve haemodynamics is mostly insufficient and needs to be supplemented by additional variables such as the body surface area and valve morphology. Furthermore, it should be noted that the VARC criteria are mainly developed for TAVI in native aortic valves [30].

Another major concern in Valve-in-Valve interventions is malpositioning of THVs [27]. On this point, procedural success was the highest after Edwards SAPIEN implantation. But a direct comparison to supraannular THVs should not be undertaken due to the mostly transapical access in the Edwards group. Medtronic CoreValve/Evolut R THV implantation required significantly more resheathings than NVT Allegra implantation. Nevertheless, in every group one valve dislocation occurred, requiring a subsequent implantation of another THV. We did not investigate the implantation depth in particular, since the vast majority of THVs were implanted in ideal positions.

In recent time, many multicentre ViV trials—the VIVA- trial for the Medtronic Evolut and the VIVALL-trial, as well as results from the SAVIV-registry for NVT Allegra THV, respectively—were published. All trials demonstrated good haemodynamic short- and long-term outcomes, accompanied by a reliable safety profile for each of the THVs. [14, 24, 25, 31]

Due to a small study population in the VIVALL trial in addition to this study, the results cannot be directly compared. However, the data implies non-inferiority of the NVT Allegra THV compared to the more broadly used Medtronic Evolut R THV, due to slightly lower gradients as seen in the trials and our study. The main difference between the two supraannular THV leading to slightly better haemodynamics of the Allegra THV might be best explained by the unique stent design with a more barrel-like frame configuration, compared to a tapered stent-design of the CoreValve/Evolut R THV[32]. Moreover, the distinct additional features of (1.) pole movement—likely translating in less leaflet stress [33], (2.) better sinus wash-out—with less blood stasis [34]and (3.) better flow patterns—with less systolic leaflet flutter [35] compared to other competitors are interesting observations. It should be noted that also after TAVI in native aortic valves good haemodynamic outcomes and a high safety profile has been described for the Allegra THV. [36]

In summary, this study demonstrates that Valve-in-Valve TAVI with the NVT Allegra THV shows at least comparable results to the more commonly used Medtronic CoreValve/Evolut R and the Edwards SAPIEN THV. Alongside with favourable short-term safety and haemodynamical outcomes, this THV might be a valid option for Valve-in-Valve interventions, even in challenging patient populations.

### Limitations

Our study has several limitations. It is a retrospective single-centre study, without an independent Core Lab for reviewing of the echocardiographic results and no matching of the patients in the three different groups. In addition, a limited number of operators performed the interventions, with different experience in ViV intervention, which naturally improved and changed over the long-lasting observation period. Obviously, different postdilation rates may have impacted the post-procedural haemodynamics. Nevertheless, at this timely stage, bioprostheses fracturing was not introduced into clinical practice. Finally, most of the Edwards SAPIEN valves have been implanted transapically, contrary to a mostly transfemoral implantation (few transaxillary n = 5 or direct aortic implantations n = 2) of the CoreValve/Evolut R and Allegra THV. Last but not least, no long-term follow-up has been performed, so only short-term safety and haemodynamical outcomes can be compared.

## Conclusions

For patients with degenerated SAVs, all three types of THV demonstrated a safe, feasible and effective treatment option. Valve-in-Valve interventions with the newer self-expanding supraannular NVT Allegra THV showed favourable haemodynamic outcomes combined with no acute mortality and no coronary obstruction. Therefore, the use of the NVT Allegra THV may be an interesting alternative to the established THV for ViV TAVI.

### Impact on daily practice

ViV interventions emerged over time to a valid alternative for redo aortic valve surgery, especially in elderly patients with failing SAV and severe comorbidities. After ViV TAVI, all three THV models showed satisfactory haemodynamic outcomes, with low mortality and complication rates, proving to be a safe and effective treatment option for patients with high risk for redo open-heart surgery. Nevertheless, supraannular THV designs seem to be the preferred devices, especially in small degenerated SAV.

### Supplementary Information

Below is the link to the electronic supplementary material.

Supplementary file1 (DOCX 369 kb)

## Data Availability

The data that support the findings of this study are available on request from the corresponding author O. Nikolayevska. The data are not publicly available due to them containing information that could compromise research participant privacy and consent.
