# Supplementary material for: Comparison of a novel self-expanding transcatheter heart valve with two established devices for treatment of degenerated surgical aortic bioprostheses
Source: Clin Res Cardiol. 2023 Apr 5;113(1):18–28. doi: 10.1007/s00392-023-02181-9 (PMC10808493; doi:10.1007/s00392-023-02181-9)
Supplement: Supplementary file 1 — Supplementary file1 (DOCX 369 kb) [file 392_2023_2181_MOESM1_ESM.docx]

**Supplement to Paper:**

Comparison of a novel self-expanding transcatheter heart valve with two established devices for treatment of degenerated surgical aortic bioprostheses

Authors:

Olga Nikolayevska^1^ MD, Lenard Conradi^2^ MD, Johannes Schirmer^2^ MD, Hermann Reichenspurner^2^ MD, PhD, Florian Deuschl^1^ MD, Stefan Blankenberg^1^ MD, Ulrich Schäfer^1,3^ MD

Department of ^1^General and Interventional Cardiology; ^2^Department of Cardiovascular Surgery, University Heart and Vascular Center, University Medical Center Hamburg-Eppendorf, Hamburg, Germany.

Department of ^3^Cardiology, Heart and Vascular Centre Bad Bevensen, Germany

Adress for correspondence:

Universitätsklinikum Hamburg Eppendorf, Klinik für Kardiologie, Martinistraße 52, 20246 Hamburg, Germany. E-Mail: [o.nikolayevska@uke.de](mailto:o.nikolayevska@uke.de)


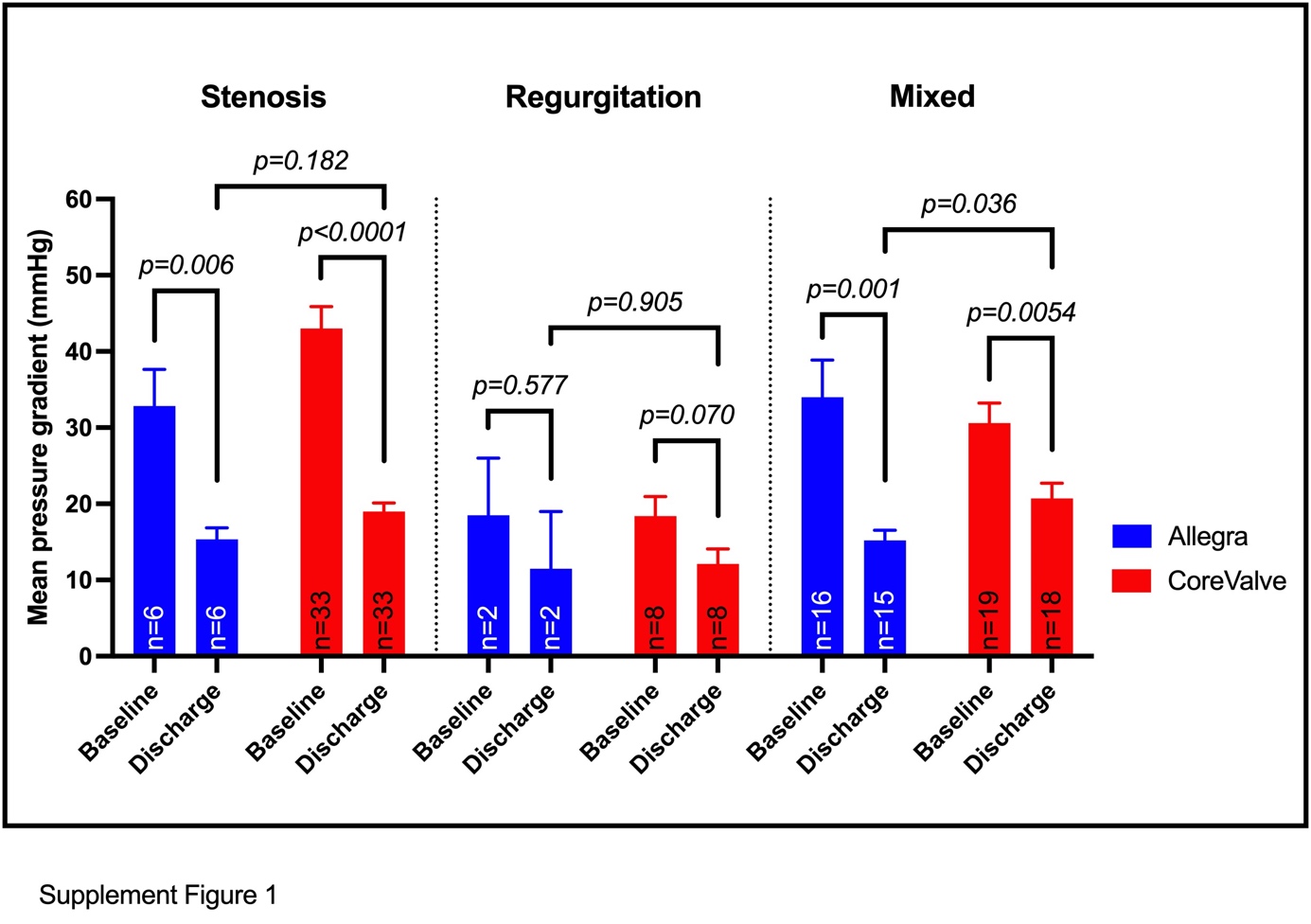


**Supplement Fig. 1**

Comparison of mean pressure gradients before and after ViV TAVI with the NVT Allegra and MTR CoreValve/Evolut R for different SAV failure modes


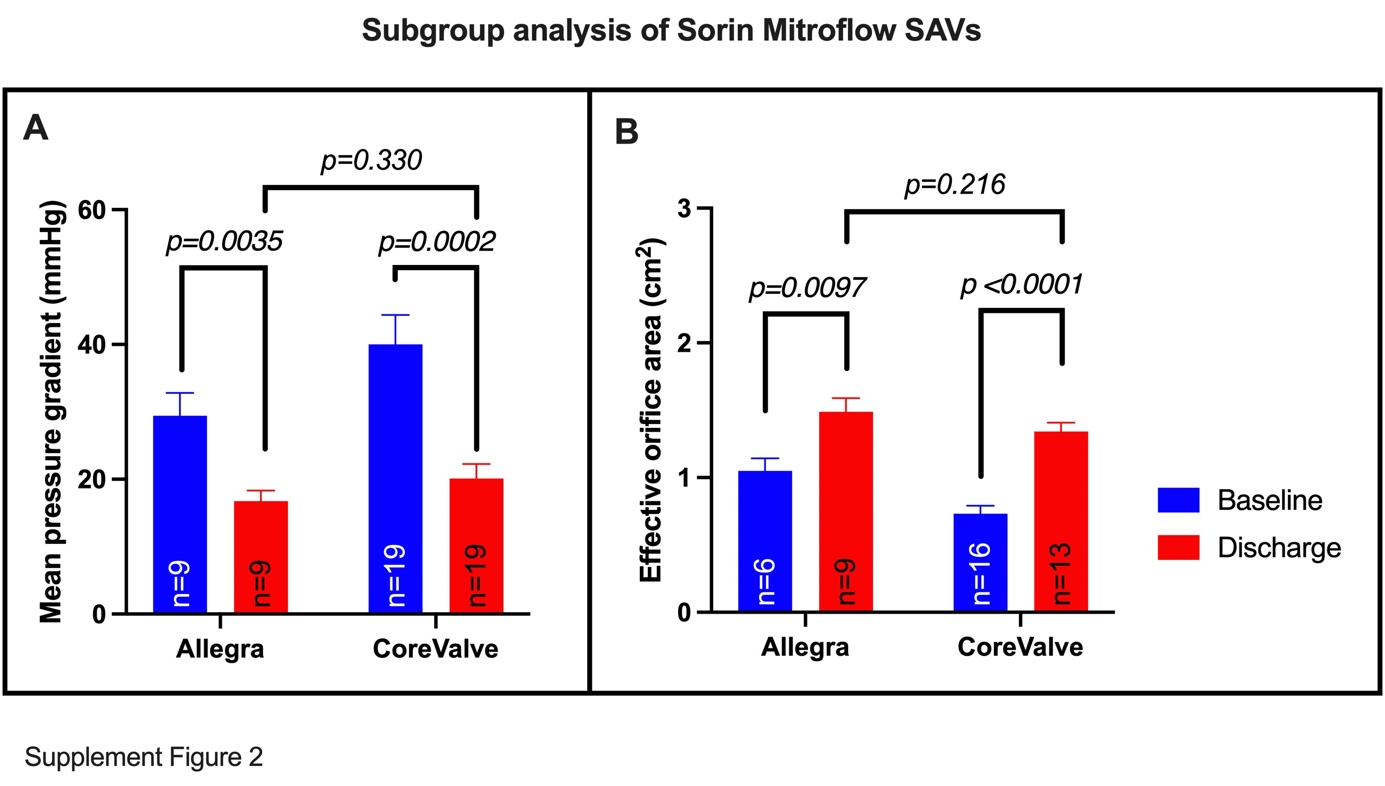


**Supplement Fig. 2** Subgroup analysis of Sorin Mitroflow SAVs

**Supplement Fig. 2a** Mean pressure gradients before and after ViV TAVI with the NVT Allegra and MTR CoreValve/Evolut R in deteriorated Sorin Mitroflow SAV

**Supplement Fig. 2b** Effective orifice areas before and after ViV TAVI with the NVT Allegra and MTR CoreValve/Evolut R in deteriorated Sorin Mitroflow SAV
